# STABILISE Technique via a Transapical Approach to Repair Residual Type A Aortic Dissection

**DOI:** 10.1055/s-0041-1729851

**Published:** 2021-09-24

**Authors:** Olivier Fouquet, Simon Dang Van, Myriam Ammi, Mickael Daligault, Christophe Baufreton, Jean Picquet

**Affiliations:** 1 Department of Cardiovascular and Thoracic Surgery, University Hospital, Angers, France; 2 MITOVASC Institute CNRS UMR 6214, INSERM U1083, University, Angers, France

**Keywords:** acute Type A aortic dissection, STABILISE procedure, left ventricular approach

## Abstract

The stent-assisted balloon-induced intimal disruption and relamination in aortic dissection or STABILISE concept is a novel endovascular strategy in Type A and Type B dissections. We report a case of Type A aortic dissection repair combining, first, an open thoracic aortic surgery with an elephant trunk procedure and, second, an endovascular treatment using the STABILISE technique via a combined transapical approach commonly used for transcatheter aortic valve implantation and a femoral pathway.

## Introduction

Acute Type A aortic dissection (AAAD) repair constitutes a surgical challenge. Residual aortic dissection carries the risk of progressive aortic dilatation, rupture, or malperfusion with the need for surgical or endovascular treatment. The two-stage repair after elephant trunk (ET) is widely used to treat disease of the thoracoabdominal aorta essentially through endovascular treatment. 1 The “Stent-Assisted Balloon Induced Intimal Disruption and Relamination in Aortic Dissection Repair (STABILISE)” concept was described in 2014, 2 the goal of which is to achieve a remodeling of the thoracoabdominal aorta with consequent obliteration of the false lumen (FL). The reapposition of the intimal flap was obtained by a balloon inflated in the true lumen to the outer layers of the aorta. 3 In some cases, the retrograde pathway, essentially iliofemoral axis, was inaccessible. An alternative route to gain endovascular access is the cardiac transapical approach. 4 We report a case of an AAAD repair combining a first stage with an ET procedure and second stage with the STABILISE technique via a transapical approach.

## Case Presentation



**Video 1** Intraoperative angiographic videos demonstrating the placement and deployment of Gore TAG stent grafts and a stent (Zenith) on Lunderquist stiff wire introduced through the left ventricular access from the elephant trunk to the celiac artery. The video shows the difficulty of catheterizing the stent through the retrograde access for the stent-assisted balloon induced intimal disruption and relamination in aortic dissection repair (STABILISE) procedure. Finally, the STABILISE technique was performed via the transapical approach with an excellent angiography result.


A 57-year-old female patient was admitted to our department for AAAD with severe aortic regurgitation and without malperfusion syndrome. The preoperative computer tomography (CT) scan showed a patent FL extending from the aortic root to the left renal artery. The dimensions of the descending thoracic aorta in zone 3 were initially at 47 mm × 49 mm.

**Fig. 1:**
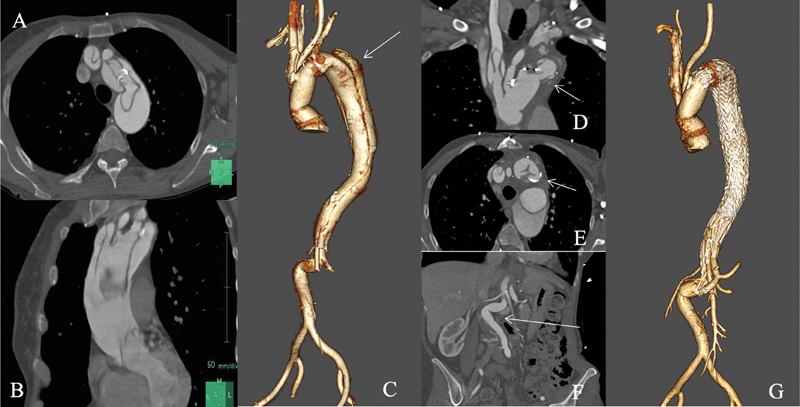
( **A, B** ) Computer tomography (CT) scan realized the day of acute Type A aortic dissection. Sagittal and transverse views showed an entry tear in zone 0 ( **A** ). The dissection process involves zones 1 and 2 and extends distally to zone 8 ( **B** ). ( **C** ) Postoperative three-dimensional reconstruction of the aorta demonstrating the aortic arch repair after elephant trunk (ET) procedure and the aortic dissection extending from the proximal descending aorta to the left renal artery. ( **D** ) and ( **E** ). Coronal and transverse, views of the postoperative CT scan demonstrating the plication of the free-floating ET prosthesis. ( **F** ). Coronal view of the postoperative CT scan demonstrating the major angulation of the abdominal aorta below the left renal artery. ( **G** ). Three-dimensional reconstruction of the final CT scan 6 months after the endovascular treatment via the transapical approach demonstrating the patency of the true lumen and the complete reapposition of the intimal flap to the wall aorta.

Replacement of the ascending aorta and aortic arch was performed under antegrade cerebral perfusion at 23°C using an ET technique due to an intimal tear involving zone 1 and a penetrating atherosclerotic ulcer in the aortic arch ( Fig. 1A, B ). This was done with a quadrifurcated prosthetic graft (Gelweave; Vascutek Woven, Renfrewshire, Scotland, diameter = 24 mm). The distal anastomosis was performed between the left common carotid and the left subclavian artery (LSA). The LSA was not connected to the branch of the prosthesis because of the interruption of the intimal tear in zone 2 and the anatomical conformity of LSA making the suture complicated. The length of the free-floating part of prosthesis in the descending aorta was approximatively 5 cm.

At 3 months, the patient presented with thoracic pain and the CT scan showed a significant increase of the proximal descending aorta diameter at 53 mm × 50 mm ( Fig. 1C ). Furthermore, a plication of the free-floating prosthesis was observed ( Fig. 1D, E ) and an angulation at 90 degrees of the abdominal aorta below the renal arteries ( Fig. 1F ).

Because of special anatomical conditions, the STABILISE technique was performed via combined transapical and transfemoral approaches.

**Fig. 2:**
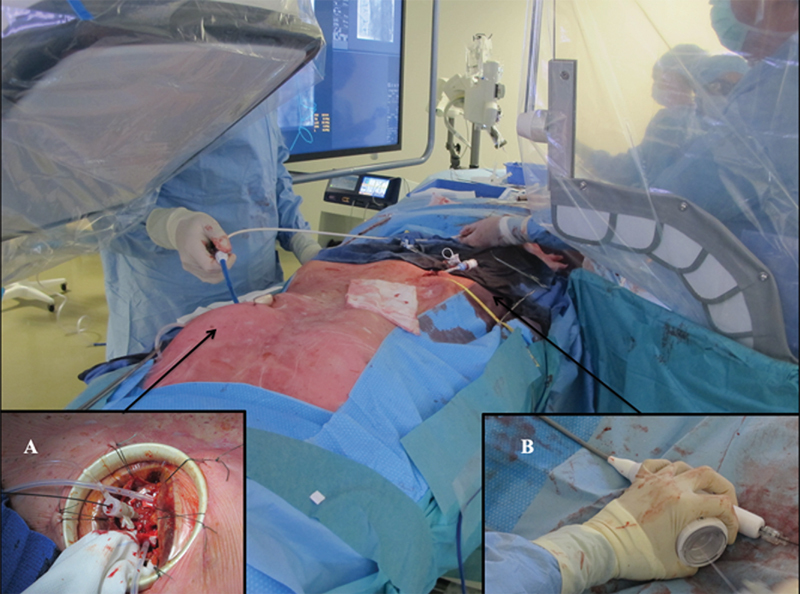
Surgical view during the stent-assisted balloon induced intimal disruption and relamination in aortic dissection repair procedure through the left ventricular (LV) approach and the right femoral access for the endoprosthesis deployment. A Lunderquist stiff guide wire was introduced through the 18-F catheter from LV access ( **A** ) to the right femoral artery ( **B** ).

The procedure was performed in a hybrid operating suite (GE Healthcare) under general anesthesia. The left ventricle (LV) was approached via a left minithoracotomy, in accordance with the technique used for transcatheter aortic valve implantation (TAVI) procedures, thereby facilitating sheath introduction and crossing the plicated prosthesis ( Fig. 2 ).

Cerebrospinal fluid drainage was performed during the procedure 1 day before the procedure with a pressure target of <15 mm Hg for 24 hours. The maximum drainage was 25 mL per hour and 40 mL for 24 hours. Monitoring of the mean arterial pressure (MAP) was performed to obtain an MAP >80–90 mm Hg. A temporary pacing wire was inserted through the right femoral vein. Due to the emergency context, the LSA was not revascularized during the second step.

A Lunderquist Extra Stiff Guide Wire (Cook Medical, Bloomington, IN) was introduced via the LV through an 18-F catheter (Sapien 3 Edwards Certitude catheter) to protect the aortic valve during its crossing with guides and because we thought it might be necessary to introduce a stent graft. The Lunderquist was caught by a lasso guide wire via the right femoral artery access. Visceral and renal branches arising from the true lumen were not catheterized during the procedure. A 22-F introducer (Gore Dryseal Flex) was placed through the femoral access.

Three Gore TAG conformable thoracic stent grafts were placed via the 22-F introducer using the Fusion technique for thoracic endovascular aortic repair and deployed in the descending aorta from the ET to the celiac trunk. A stent (Zenith Dissection Endovascular Stent, diameter = 36 mm and length = 180 mm) was then deployed from the distal endoprosthesis up to the angulation of the aorta.

**Fig. 3:**
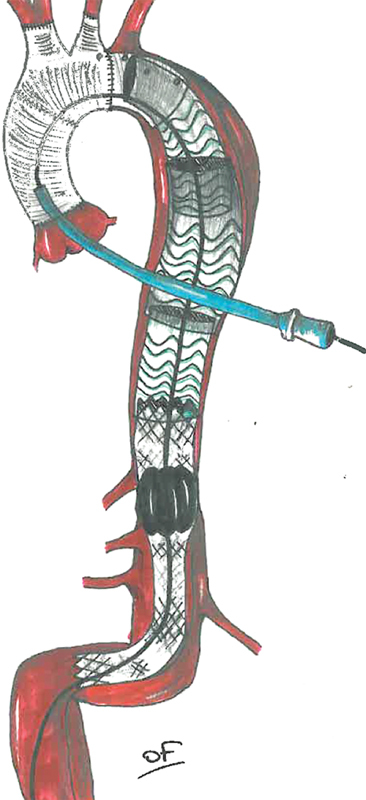
A diagram of the stent-assisted balloon induced intimal disruption and relamination in aortic dissection repair technique via the left ventricular access.

Catheterization of the stent was unsuccessful through the retrograde pathway with the trilobe balloon catheter (Gore Medical, Flagstaff, AZ) due to the aorta angulation. The STABILISE procedure was finally performed through the transapical LV access using the trilobe balloon on the Lunderquist extra stiff wire ( Fig. 3 ). The balloon was manually inflated under fluoroscopic control and overlay of fusion images corresponding to nominal diameter of the aorta. An angiographic control was performed via the LV apex ( Video 1 ; available in the online version).

The patient was discharged on postoperative day 10 without complications. CT scan at 6-month follow-up confirmed complete realignment of the aorta with true lumen patency, complete reapposition of the intimal flap to the aortic wall, and patency of all aortic branches ( Fig. 1G ). Revascularization of LSA by a carotid–subclavian bypass was performed at 8 months due to a vestibular syndrome.

## Discussion

To our knowledge, this is the first reported case in the literature of AAAD repair with the STABILISE technique through a transapical approach after previous ET procedure. Using a frozen ET during the initial procedure was probably the best alternative. The STABILISE concept was first described by Hofferberth et al 2 and aimed to achieve FL restoration and immediate restoration of uniluminal aortic flow by a trilobe balloon catheter inside a compliant stent. It was initially proposed for Type B aortic dissection with an intimal tear in the descending aorta. 5 Exclusion criteria for this technique included complete FL thrombosis, aorta diameter of >40 mm, and dissection limited to the thoracic descending aorta. 5

The technique has now been performed in AAAD 6 with excellent midterm results of aortic remodeling. The use of transapical approach to treat aortic dissection was published a few years ago. 7 The complexity of our case was exacerbated by the plication of the free-floating prosthesis, the residual dissection of the brachiocephalic artery, and the major angulation of the abdominal aorta.

In conclusion, the transapical approach, commonly used in the TAVI procedures, offers quick and safe access for endoprosthesis deployment and the STABILISE technique when the retrograde approach is problematic.
